# Supplementary figures and images for: Association between Epstein-Barr virus reactivation and severe malaria in pregnant women living in a malaria-endemic region of Cameroon
Source: PLOS Glob Public Health. 2024 Aug 12;4(8):e0003556. doi: 10.1371/journal.pgph.0003556 (PMC11318859; doi:10.1371/journal.pgph.0003556)

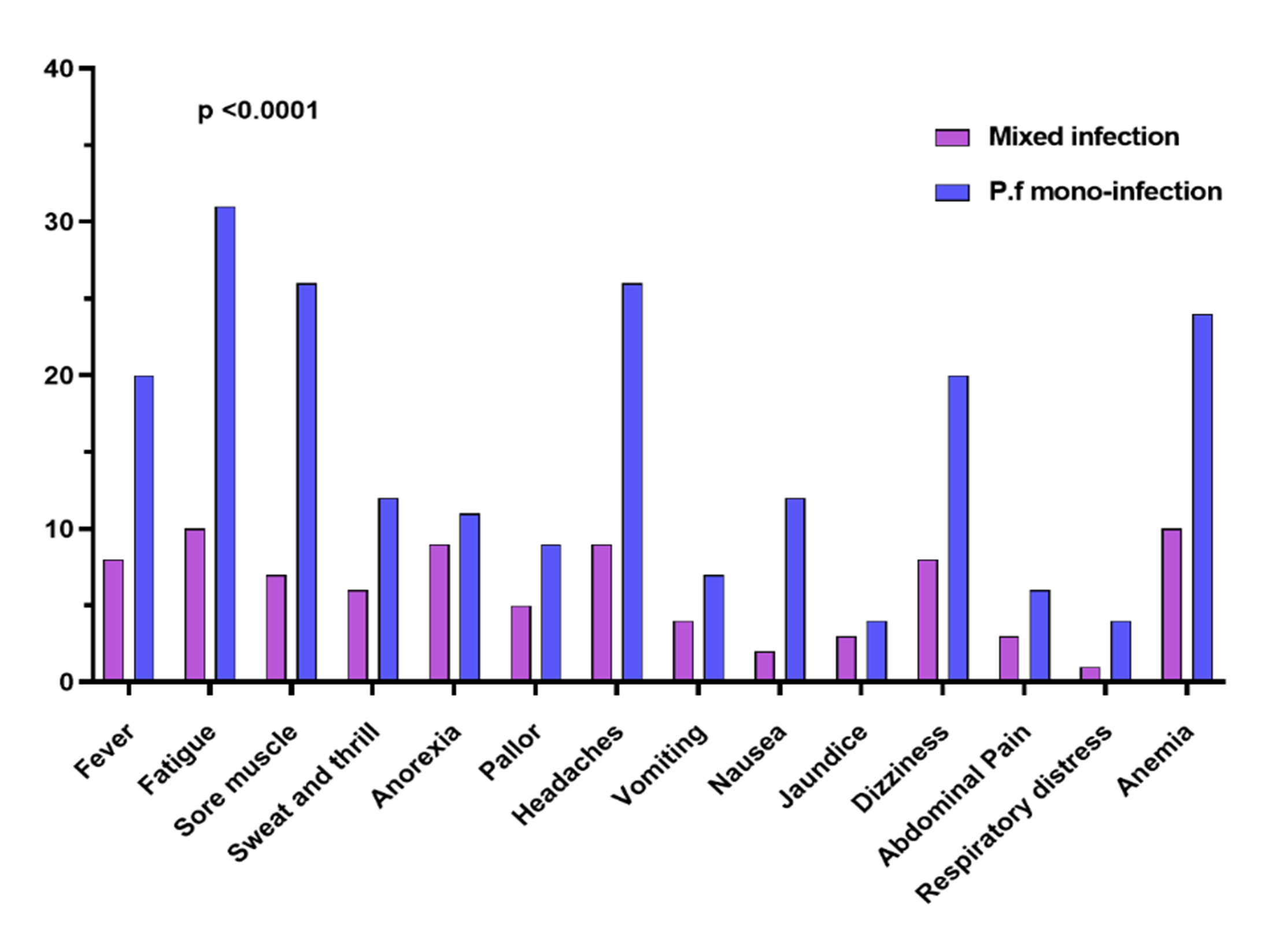

Supplement: S1 Fig — The Mann-Whitney U test was used for statistical analysis. (TIF) [file pgph.0003556.s001.tif]

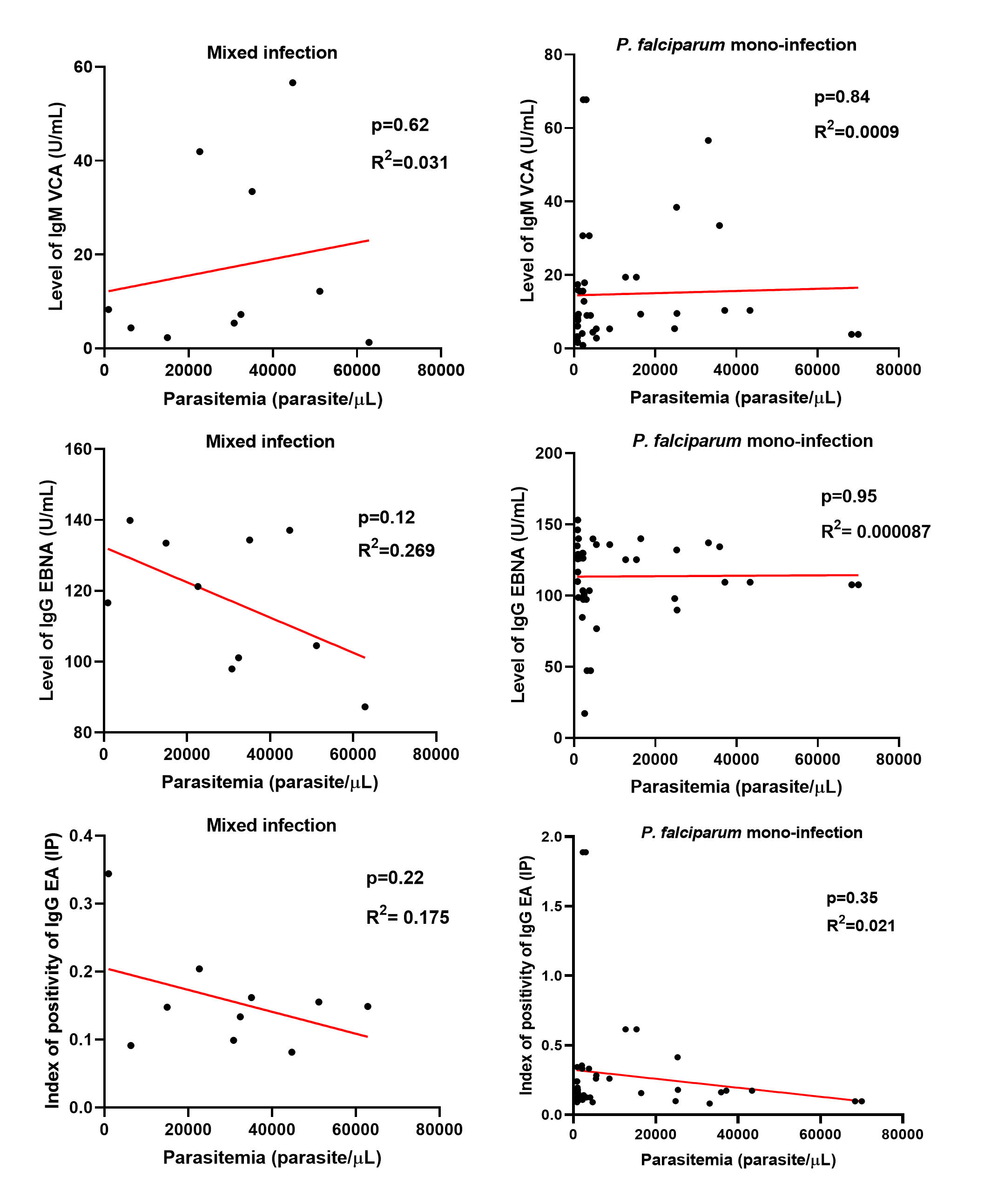

Supplement: S2 Fig — Statistical analysis was done by linear regression (R2). (TIF) [file pgph.0003556.s002.tif]
