# Supplementary material for: Association between Epstein-Barr virus reactivation and severe malaria in pregnant women living in a malaria-endemic region of Cameroon
Source: PLOS Glob Public Health. 2024 Aug 12;4(8):e0003556. doi: 10.1371/journal.pgph.0003556 (PMC11318859; doi:10.1371/journal.pgph.0003556)
Supplement: S1 Appendix — (PDF) [file pgph.0003556.s003.pdf]

S1 Appendix : Questionnaire

N° Anonym ☐

Study site ------------------------------------

1) Demographic characteristics

Frist name (s) and surname (s): ------------------------------------

Age: ☐

Weight: --------- Height: --------- Body mass index (Kg/m^2):---------

Marital Status: Married ☐ Single ☐ In relationship ☐ Divorced ☐

Maternal Education: Less than High School ☐ High School Graduate ☐ College ☐

Quarter ------------------------------------

Environment: Rural ☐ Urban ☐ Swamp ☐

Maternal race/Ethnicity ------------------------------------

Profession ------------------------------------

Employment pattern: physical labor ☐ Mental labor ☐ Unemployment ☐

Parity: Primigravidae ☐ Multigravidae ☐

Gestational age: 1^{rst} Trimester (<14s) ☐ 2nd Trimester (14-27s) ☐ 3rd Trimester (≥28s) ☐

Date of last menstrual period: ☐

Maternal income: ------------------------------------

2) Clinical Data

Fever (≥ 38 C) ☐ Sleeping too much ☐

Fatigue ☐ Sore muscle ☐

Sore throat ☐ Cough ☐

Headaches ☐ Nausea ☐

3) Health Behaviours

Tabaco use: Yes ☐ No ☐

Illicit substance abuse: Yes ☐ No ☐

Alcohol drink: Yes ☐ No ☐

Sexually transmitted infection: Yes ☐ No ☐

Antiviral drugs use or Antidepressant: Yes ☐ No ☐

4) Indicators of Severe Malaria

Malaria frequency: (per Month) ☐ (per Year) ☐

Severe Anaemia (Hb < 5g/dl and Ht < 15%) ☐ Anorexia ☐

Diaphoresis ☐ Abdominal pain ☐

Jaundice ☐ Pallor ☐

Diaphoresis ☐ Dizziness ☐

renal failure ☐ Sweats ☐

Cerebral malaria ☐ Impaired consciousness ☐

Convulsions ☐ Respiratory distress ☐

Use of preventive antimalarial drug: Yes ☐ No ☐

Mostiquo net: Yes ☐ No ☐

**Laboratory Findings**	**Results**
Parasitemia (parasites/µl)	
Haemoglobin (11g/dl)	
White blood cells (10^9/l)	
Red blood cell (10^{12}/l)	
Hypoglycemia (< 2,2 mmol/L)	

5) Risk factors of EBV reactivation and associated symptoms

Stress: Yes ☐ No ☐

Anxiety: Yes ☐ No ☐

Smoking: Yes ☐ No ☐

Fatigue: Yes ☐ No ☐

Heart palpitations: Yes ☐ No ☐
