# Supplementary material for: Association between Epstein-Barr virus reactivation and severe malaria in pregnant women living in a malaria-endemic region of Cameroon
Source: PLOS Glob Public Health. 2024 Aug 12;4(8):e0003556. doi: 10.1371/journal.pgph.0003556 (PMC11318859; doi:10.1371/journal.pgph.0003556)
Supplement: Attachment — Submitted filename: Response to Reviewers.docx [file pgph.0003556.s004.docx]

To,

**Prof. Mathieu Nacher**
Academic Editor
PLOS Global Public Health

Dear Prof. Mathieu Nacher,

Thank you very much for considering our research article entitled **“****Association between Epstein-Barr virus reactivation and severe malaria in pregnant women living in a Malaria-endemic region of Cameroon*”*** by Djuidje Chatue et al., for revision. The authors appreciate and extend thanks for the kind consideration and thorough review of this manuscript that allowed us to revise and improve the manuscript. Keeping the reviewers helpful and validly raised concerns and questions in mind we modified and improved the manuscript.

We have addressed the questions raised or corrections suggested by all the reviewers point by point. Please find the details below.

Best wishes and regards

DJUIDJE CHATUE Ide Armelle

Ph.D. Student in Medical Microbiology, University of Yaounde I

**Journal Requirements:**

1. Please update your online Competing Interests statement. If you have no competing interests to declare, please state: “The authors have declared that no competing interests exist.”

**Response:** The declaration of competing interests has been updated online.

2. Please provide separate figure files in .tif or .eps format only and remove any figures embedded in your manuscript file. Please also ensure that all files are under our size limit of 10MB. You may leave the figure captions or legends in the manuscript.

**Response:** All figures have been removed in the manuscript and upload to the Preflight Analysis and Conversion Engine (PACE) digital diagnostic tool.

3. We notice that your supplementary figures are included in the manuscript file. Please remove them and upload them with the file type 'Supporting Information'. Please ensure that each Supporting Information file has a legend listed in the manuscript after the references list.

**Response:** The supplementary figures have been removed in the manuscript and upload with the supporting information file.

4. We do not publish any copyright or trademark symbols that usually accompany proprietary names, eg (R), (C), or TM (e.g. next to drug or reagent names). Please remove all instances of trademark/copyright symbols throughout the text, including ® on pages 9, 10, 11 and 12.

**Response:** All copyright and trademark symbols have been removed on these pages.

5. Some material included in your submission may be copyrighted. Figure 1: please (a) provide a direct link to the base layer of the map (i.e., the country or region border shape) and ensure this is also included in the figure legend; and (b) provide a link to the terms of use / license information for the base layer image or shapefile. We cannot publish proprietary or copyrighted maps (e.g. Google Maps, Mapquest) and the terms of use for your map base layer must be compatible with our CC-BY 4.0 license.

**Response:** The copyright and the Creative Common license of the figure 1 and the direct link to the base layer of the map has been provides in the manuscript.

**Reviewer(s)’ Comments to Author:**

**Reviewer #1**

**Comment:** This article raises an important question on which there is very little data, that of the relation between severity of malaria during pregnancy and reactivation of EBV.
The work is well conducted, original and well written.

I congratulate the authors for the quality of the study and of the paper, which will be very useful for the malarialogist community.

However, the article would benefit from lighter coverage of the secondary issues and stronger emphasis on the main question - the link between severity and EBV reactivation - which is given little attention in the results and discussion. Thus, the number of patients with EBV reactivation and severe malaria is not clearly presented. The main result is presented in reverse, even in the abstract, presenting the link between EBV reactivation and uncomplicated malaria. It would be much clearer to present it the other way round: link between EBV reactivation and severe malaria, presenting the numbers, which are likely to be small.

**Response:** We extend our sincere appreciation to the reviewer for recognition of our effort in this work. the required corrections have been made in the manuscript. The number of patients with EBV reactivation and severe malaria has been clearly presented. The OR was calculated taking severe malaria as the outcome.

**Other major correction and Comments**

1-Abstract: Present the main result with regards to severe malaria.

**Response:** We thank the reviewer for recognizing our efforts in understanding the relationship between the severity of malaria during pregnancy and EBV reactivation. The presentation of the main result has been corrected in the manuscript.

2- Introduction: L93: and children?

**Response:** EBV clinical symptom in children has been added in the manuscript. However, we want to mentioned that most clinical symptoms appear in adolescents and young adults. Since our study focuses on pregnant women, we also wanted to present the consequences of EBV reactivation during pregnancy.

3- L94: “can” instead of “cause”

**Response:** “can” has been added to the sentence. Removing “cause’’ would change the meaning of the sentence.

4- L96: “decrease” instead of suppression

**Response:** The requires correction has been made as suggested.

5- L120-121: not clear: what do you mean with “naturally clear the parasite”? without treatment? Are there situation when a pregnant woman with malaria is not treated? Even asymptomatic? Please clarify.

**Response:**  We acknowledge the reviewer for his concern about the declaration ''naturally clear the parasite''. We have added references in the manuscript to make the readers understands better.

In malaria- endemic areas, people develop over time and with repeated exposure, an acquired natural immunity to *Plasmodium spp*. The latter involved the antibody response. These antibodies can offer protection against high-density parasitemia and severe clinical manifestations. Several distinct antibody functional mechanisms have been defined in *Plasmodium* parasite clearance (DOI: [10.1002/JLB.5MR1021-537R](https://doi.org/10.1002/jlb.5mr1021-537r)). Spontaneous clearance of *P. falciparum* infections in the absence of antimalarial drug treatment has been demonstrated in a range of transmission settings and confirmed by sensitive molecular diagnostics (<https://doi.org/10.1016/j.pt.2022.02.005>).

However, there are no situations in which a pregnant woman with malaria goes untreated. Any confirmed case of malaria during pregnancy is subject to appropriate treatment, as recommended by the WHO. In addition, in malaria-endemic areas of Africa, intermittent preventive treatment with sulfadoxine-pyrimethamine (SP-IPTp) is recommended for all pregnant women during their first or second pregnancy. Treatment should begin during the second trimester, and doses should be administered at least 1 month apart, to ensure that at least three doses are received (**Reference:** <https://www.who.int/tools/elena/interventions/iptp-pregnancy>).

6- L124: what do you mean with “possible outcome”? please specify

**Response:**  We have clarified this sentence in the manuscript. Here we wanted to evaluate the impact of EBV reactivation on the health conditions of malaria patients, if EBV reactivation worsen the clinical symptoms of malaria in pregnant women and lead to severe unexpected outcome like cerebral malaria, coma, abortion, or even affect their newborn (in a future study).

7- Material and method: L138: low-cost: care are not free for pregnant women in Cameroun?

**Response:** Care is not free for pregnant women in Cameroon but the costs of antenatal consultations, laboratory exams and ultrasounds are cheaper in public health facilities than private structure.

8-L154-155: please clarify what you mean with “participants approached the hospitals”: this refers to reason of consultation? Or is it an inclusion criteria? If yes modify. Did you included only women consulting for fever?

**Response:** No, this is not a reason for consultation or an inclusion criteria. It is recommended to every pregnant woman to approach hospitals or any health structures for a better pregnancy management and follow-up. For this study, we took advantages of antenatal consultations to recruit pregnant women who manifest malaria associated symptoms, such as fever (temperature ≥ 38°C) accompanied by chills, asthenia, fatigue, headache, sore throat, sore muscle, sweat and thrill, pallor, jaundice, dizziness, abdominal pain and respiratory distress and other inclusion criteria mentioned in the manuscript. So, it is normal for pregnant women to approach hospital.

9- L157-162: combine non-inclusion and exclusion criteria to avoid repetition, or explain the difference (timing? it the serological results are known before consent?)

**Response:** Non-inclusion and exclusion criteria have been combined them to avoid duplication as the reviewer suggested. Some serological results were known prior to consent, but for those that were not, serological testing was carried out after consent.

10- Why did you excluded women with antibiotics?

**Response:** This manuscript presents our preliminary result of our research work; the second part evaluates the activity of cytokines during co-infection EBV reactivation and malaria. It has been reported that certain antimicrobials, such as antibiotics, can modify host immune and inflammatory responses in both in vivo and in vitro. This could influence the inflammatory cytokine response during malaria and trick our results. Here we wanted to eliminate the factors that could have caused confusion in this study. (DOI: https://doi.org/10.1128/aac.40.6.1366).

11- How did you defined depression?

**Response:** Using a well-structured questionnaire, the depression was defined by the use of antidepressants, and the result was reported as Yes/ No.

12- L166: “all” instead of “various”?

**Response:** The required corrections have been made in the manuscript.

13- L170: how did you proceed to the random sampling? Please specify

**Response:** The participants were chosen randomly, and each participant had the same chance of being selected. During the antenatal consultations, we have selected and interviewed pregnant women in waiting rooms, as the consultations were taking place simultaneously.

14- L173: P” is the prevalence of EBV and malaria coinfection among pregnant women in Kenya, which is 64% [31].”: it seems very high, it is among women consulting for fever? If yes specify

**Response:** This prevalence is not among pregnant women consulting for fever. In this study, the author included Kenyan pregnant women of all gravidities, less than 30 weeks gestation, with a normal blood count, HIV-negative, residing within a 10 km radius of the hospital and willing to return to the clinic for follow-up clinical procedures and laboratory tests. Pregnant women enrolled showed no signs of symptomatic malaria during follow-up visits (DOI: 10.1007/s10995-014-1546-4).

15- L177: please provide the questionnaire as a supplementary material

**Response:** The Questionnaire has been provided as a supplementary material.

16- How did you defined the bednet use? In your results it is presented yes/no: what was the question? The night before the inclusion? Usually? Everyday?

**Response:**  Participants were asked during the interview if they used or owned a mosquito net. The results are presented as yes/no. Yes, for those who had a net or used it in the evening at bedtime and No represented those who didn't have a net or didn't use one.

17- Idem for IPT: how is defined yes or no? according to the trimester of pregnancy? At least one dose? What if very early pregnancy?

**Response:**  During the interview, participants were asked if they had taken IPT since the start of their pregnancy (if so, the number of doses was also reported). The results were presented as yes/no. Yes, for those who had taken at least one dose during pregnancy and no for those who had not taken IPT.

18- How did you measure anxiety?

**Response:**  Using a questionnaire, the anxiety was measured by asking participants if they had been anxious or in a situation of extreme worry in the last few days. The results are presented as yes/no.

19- L192: In my opinion, all the details concerning microscopy and PCR are too detailed. They should be grouped together in a paragraph of no more than 10 lines, with references to the methods used and the detection threshold.

Table 1 should be deleted.

**Response:** We thank the reviewer for providing valuable insights. We have synthetized each part as short as possible and table 1 has been deleted. It was important for us to detailed the microscopy so that readers can understand how the thick drops were done to detect *Plasmodium* parasite forms. It will also help the readers to understand how parasitemia was calculated and classified. Furthermore, the definitions of severe malaria were also included. The methodology of the PCR has been detailed because we have made some modifications to the amplification conditions and master mix composition. All this information is essential in our methodology and helpful for the readers.

20- However, please explain why you did the PCR on filter paper when you have whole blood? Was it sent to another laboratory? It would have been easier to perform the PCR directly on the blood and increase sensitivity. Please explain why.

**Response:** We recognize the importance and the advantage of performing the PCR with the whole blood as the reviewers raised the concern. Indeed, we did not have the necessary resources and technical facilities to carry out Nested PCR in our home Laboratory in Cameroon but thanks to the mobility fellowship programme “We-Star” awarded to Djuidje Chatue, the blood samples were stored on Whatman paper and the DNA was extracted and transported to the Malaria Biology Laboratory at ICGEB in New Delhi, India for molecular analysis.

21- L254-268: idem too detailed in my opinion, to be summarized
Please add the definition of severe malaria++

**Response:** We have deleted details on the ELISA technique for EBV diagnosis and we have made it short. The antibody detection thresholds and the table defining the clinical status of EBV have been maintained in the manuscript. The definition of severe malaria has been included in the section Malaria Diagnosis.

22- Results: L287: and CMV? inclusion criteria

**Response:** For CMV, we referred to the patient's health Book.

23- L288-293: redundant with flow chart, could be deleted

**Response:** The required correction has been made in the manuscript

24- Fig2: remove “ethical clearance…” from the title. The last line is very interesting but should be presented as a tree. Table 3: please define “maternal income”

**Response:** The first line of the figure 2 has been deleted and the last line has been restructured as a tree as suggested. We can’t to define maternal income because the participants were reluctant to give me their exact monthly income or an estimation. That's why we've classified the answers as low, middle and High, but none of the participants had declared high monthly incomes. In terms of occupation, 41 (18.63%) of the participants were farmers and 69 (31.36%) had no employees.

25- L312: you did not present gestational age as a continuous variable, and you did not present BMI, please correct. Table: please correct the table footer calls

**Response:** The table footer has been corrected.

26- As mentioned in the method, the use of IPT and LLIN must be defined, either in the method or here in the form of a footnote.

**Response:** The use of IPT and LLIN has been defined in the method section.

27- L328: must be deleted

**Response:** The required correction has been made in the manuscript.

28- Fig3: in my opinion, should be deleted as it does not provide any useful information

Fig4: would be more interesting to describe these % in the study population and not % of plasmodium positive

**Response:**  Fig 3 has been deleted. In the Fig 4, the percentages of *Plasmodium* *spp* have been described in the study population as suggested by the reviewer.

29- L343: first sentence to be removed

**Response:**  The required correction has been made in the manuscript.

30- L347 and above: the number at the beginning of a sentence must be in letters, otherwise it must be rephrased. Parasitemia in pregnant women: could provide the median parasitemia among women with severe malaria vs uncomplicated malaria?

**Response:**  The numbers at the beginning of the sentence have been rewritten in letters and parasitemia in pregnant women among women with severe malaria vs uncomplicated malaria has been provided.

31- L361-364: the % presented concern which population? IT

**Response:** The percentages presented concern the malaria population.

32- L367: provide the numbers N++

**Response:** The number N have been provided in the manuscript.

33- L379: “Symptoms of EBV infection were similar in patients with EBV reactivation or latent infection.” What do you mean? Please clarify

**Response:**  We meant that the statistical analysis could not show a significant difference between the clinical symptoms of reactivated and latent EBV. The symptoms of reactive EBV infection appeared to be statistically similar to those of latent infection. Perhaps because they were very not specific to the infection? The symptoms reported in our study population were the following: fatigue, heart palpitations, cough, fever, sore throat, angina.

34- L379: The sentence has been clarified.

35- “Association between EBV reactivation and malaria severity in pregnant women”: this is the main result of your study but the results are not clear. Please provide the n/N for each category (severe/non severe malaria, EBV reactivation y/n, what did you do with the woman who had no EBV?).

And please calculate the OR with “severe malaria” as the outcome: it would be much clearer and help the reader understand your main result. This should also be included in your abstract.

**Response:** The required correction has been made. The n/N were provided for each category and the OR was recalculated. We had only one participant with no EBV and one with acute EBV. We excluded them for the analysis of the association between EBV reactivation and malaria severity.

36- Discussion: Start the discussion with a brief summary of your results (this comes too late, L514). And point out that this is a first investigation of this question (which you say L519 > to move). Then present the limitations/biases: your sample of EBV reactivation/malaria is small (N not clearly presented, 12?). But even with this limitation, you have important results; you just need to specify the bias so that the reader can interpret the relevance of your work.

**Response:** We thank the reviewer for the thorough analysis of our work and for highlighting the mistakes. All the required correction has been made in the discussion section. L514-549 was moved to the beginning of the discussion and the small sample size for EBV/malaria reactivation has been added to the limitations.

37- L441: not relevant. L459-460: not relevant.

**Response:** L441 and L459-460 have been deleted.

38- L467 and above: I don't think it's relevant to this article. There's no link with the objective of the study, so it's difficult for the reader to see the point. L472-477: idem: in your study they were all infected by Pf, at least; so not relevant

**Response:** L467 and above: has been deleted. L472-477: has been deleted

39- L501-503: to be placed in the limitation. L510-513: It's a complex question that is not related to your main objective. I suggest you delete it to simplify your text. Otherwise, it would deserve much more explanation.

**Response:** L501-503: has been placed in the limitation. L510-513: has been deleted.

**Reviewer #2**

**Comment:** The authors evaluated the proportion of cases of severe and non-severe malaria in pregnant women randomly selected in 3 centres in Cameroon according to the presence of EBV reactivation. They showed that women with EBV reactivation were more often affected by severe malaria. A link was also established between EBV reactivation profile and parasitaemia.

The diagnostic method seems robust and the work useful for confirming the hypothesis of a link between reactivation and severe malaria in pregnant women.

The study initially included 220 patients, but in the end only 51 had malaria and the number of patients with reactivation and malaria was only 12, which is a very small number for further analysis especially a multivariate analysis was not done here which is somechat problematic considering the confounding factors of either having positive EBV serology or severe malaria. Besides only serological analyses of EBV were done and no EBV viral load was obtained which could have been informative to ascertain the EBV reactivation profile and avoid possible false positive serological reactivation profile due to immune activity caused by malaria especially severe malaria. This should be discussed.

The article is well written and clearly formulated. Some minor corrections are needed to the English version. However, the study suffers from a number of limitations and several points need to be corrected to improve clarity and conciseness.

**Response:** We extend our sincere appreciation to the reviewer for recognition of our efforts to show the link between EBV reactivation and severe malaria in pregnant women. We recognized that the main limitation of this study is the low number of participants with EBV reactivation and malaria despite that the methodology was reliable, reproducible and well documented. Indeed, the selection criteria for participants were rigorous, as we wanted to obtain quality results. This led to the exclusion of many patients with other infections that could confound the interpretation of our results. We performed univariate logistic regression rather than multivariate analysis. Concerning EBV, we had 4 groups: EBV reactivation (29/220), Latent EB (189/220), seronegative EBV (1/220) and acute EBV (1/220). For malaria, we had 2 groups: severe malaria (20/51), uncomplicated malaria (31/51). There were 169 malaria-free patients. Among those with EBV reactivation, 12 were infected with malaria (8 with severe malaria and 4 uncomplicated), while among latent EBV patients, 38 had malaria (12 with severe malaria and 26 uncomplicated). These groups were used in univariate analysis the association and determine the OR with severe malaria outcome.

We performed serological analyses for EBV diagnosis to detect specific EBV antibodies (IgM VCA, IgG EA and IgG EBNA). These antibodies enabled us to define the serological status of each patient (acute infection, past, recent, or reactivated infection). This technique is widely used and recommended, as PCR cannot differentiate between acute, past or recent infection. However, serology also has its drawbacks, as mentioned by the reviewer, namely the presence of serological patterns whose interpretation can be equivocal. On the other hand, the PCR can be used as a complementary tool for DNA quantification, which also reveals the status of a reactive infection thanks to a copy threshold). It is well known that recurrent attacks of malaria disrupt EBV latency and induce reactivation, but in our study only 8 patients with severe malaria had EBV reactivation. The main cause of EBV reactivation in pregnant women in the study is unknown. No risk factors such as stress, anxiety, alcohol or tobacco consumption were associated with EBV reactivation. We believe that pregnancy in itself may have favored EBV reactivation from latency due to the diminished cellular immune response.

**Others majors correction and Comments.**

1- Abstract: Please rephrase « were less likely to develop uncomplicated for more likely to develop complicated malaria which is easier to understand »

**Response:** We appreciate the reviewer's valuable feedback. The sentence has been rephrased and the link of EBV reactivation with regards to the severity of malaria has been clarified (L64-66).

2- L77 – 78 please remove

**Response:** This data presents the burden of malaria worldwide and the situation in Cameroon, where the study was carried out. We think is an important information.

3- L 88 due to poor health could you add some reference here?

**Response:** Two references have been added (L87-88).

4- L 99 is useful to add so much details here?

**Response:**  L99-104 has been deleted.

5- L 124 The hypothesis of the work should formulate more clearly. Assessing if EBV reactivation increases the level of severity in malaria attack in pregnant women.

**Response:**  We thank the reviewer for the thorough analysis of our work and for highlighting the misunderstanding. The required correction has been made.

6- Methods: It is not very clear why HBV and HVC were exclusion criteria in this work, please clarify this point

**Response:**  HBV and HCV were considered as exclusion criteria because they may influence the immune response against malaria. In fact, this manuscript presents the results of the first part of our study, the second part evaluate the biosignature of cytokines during EBV reactivation in malaria patient. Given that HBV induces a robust pro-inflammatory type 1 (Th1) immune response, which is important for *Plasmodium* clearance, but is also implicated in disease severity. This could be a bias for the usability of our study. We wanted to eliminate factors that might have caused confusion in this study.

7- Also why did anti histaminic or confirmed depression were considered as potential bias?

**Response:** Studies have shown that women suffering from depression have a higher prevalence of Epstein-Barr virus reactivation, probably due to increased stress (**Reference:** Haeri, Sina MD, MHSA; Johnson, Nell MD; Baker, Arthur M. MD; Stuebe, Alison M. MD; Raines, Christena NP; Barrow, David A. PhD; Boggess, Kim A. MD. Maternal Depression and Epstein-Barr Virus Reactivation in Early Pregnancy. Obstetrics & Gynecology 117(4): p 862-866, April 2011. | DOI: 10.1097/AOG.0b013e31820f3a30). For antihistamines, it has been revealed that some allergy medications can induce EBV viral reactivation (<https://presse.inserm.fr/certaines-allergies-medicamenteuses-sont-des-reactions-a-des-virus-dormants/14842/>). As these are confirmed factors that can induce reactivation, we have excluded them in order to study and identify other poorly documented factors such as stress, anxiety, alcohol consumption, age, parity, and gestational age.

8- The sample size determination should be assessed by a specialist

**Response:** Here, we have used Cochran's formula for a large proportion of the population and the reference has been added.

9- The risk factors of EBV reactivation are based on what references? please add some information here

**Response:** The risk factors of EBV reactivation are based on previous study available in the literature review. References have been added in the manuscript.

10- Methods: regarding parasitaemia levels would be possible to convert for % of parasitaemia for better understanding? Is the level of EBV antibodies usely done and is it based on quantitative measures? I am not very familiar with it, please clarify

**Response:** The number of parasites was counted per 200 leukocytes, and the parasite density was calculated as described by the WHO, assuming a total WBC count of 8,000 cells/µL of blood. To convert to a percentage, the number of *Plasmodium*-infected red blood cells should be counted over the total number of red blood cells in at least 3 fields but unfortunately patient’s blood was dried and stored on Whatman paper.

Yes, the anti-EBV antibody level is a quantitative measure that can be determined using several techniques such as the ELISA, used in our work. It is not commonly measured. We wanted to quantify anti-EBV antibody levels to assess the correlation between antibody levels and parasitemia, as well as the severity of malaria.

11- Fig 2 it seems a bit odd for med that subjects agreed to participate before being assessed for eligibility in the selection process.

**Response:** In fact, before participants enrolment, they objectives, benefits and risks of the study was presented to them. Informed consent was then obtained, and participants were interviewed to verify eligibility criteria. Serological status of patient was collected in their health book but for unknown serological status, we performed HIV, HCV, HBV and Toxoplasmosis tests.

12- L285 What does « symptomatic » refer to here? symptomatic for pregnancy? or symptomatic for malaria suspicion only confirmed in 51 patients- in the end?

**Response:** We value the insightful comments provided by the reviewer. “symptomatic” refer to symptomatic for malaria suspicion.

13- L302 this part should be in methods section

**Response:** This has already been detailed in the methods section. However, we have rephrased the first introductory sentence of this section.

14- Why the authors report the fact that subjects were married? is it useful here? please justify. Again, regarding the ethnic group how does it matter for the research purpose? please clarify.

**Response:** Marital status and ethnic group are part of the socio-demographic data of the study population. Generally, in descriptive or cross-sectional studies, it's important to specify and describe the population we're working on. In fact, it's been shown that this data often enables the authors to discuss the results obtained in comparison with other studies.

15- Definitions of low and middle income should be given in the manuscript

**Response:** Incomes were defined qualitatively, as the participants did not want to divulge exact or approximate amounts of their income. We asked them whether their income was low, medium or high, depending on their occupation. (See questionnaire in supporting information).

16- Table 4 In my opinion the authors have presented the results with sums per line rather than per column, which makes reading the results more complex and less informative.

**Response:** We appreciate the constructive feedback from the reviewer regarding the table 4. The required correction has been made in the manuscript.

17- L 330 the details of positivity for non-*falciparum* species should be given after mentioning the results of coinfection.

**Response:** We have chosen to put the results of *Plasmodium* species identification after the prevalence of malaria to follow the same order of idea as our specific study objectives and methodology. Firstly, present the results of malaria diagnosis, then EBV, then coinfection. This is for ease of reading.

18- 343. this should be put in methods section

**Response:** This sentence has been deleted.

19- 352 Please could you clarify if 40% of severe forms were due to mixed infections? Considering that mixed infections only represented 20% of the forms it seems that having mixed infections was associated to the occurrence of severe malaria. The authors seem to emphasize L 352-353 that most of cases were due to monoinfection. If 40% of severe forms come from only 20% of the whole cases I would emphasize more on this fact. Please clarify

**Response:** We thank the reviewer for the thorough analysis of our work and for highlighting the misunderstanding. Of the 51 pregnant women with malaria, 20 presented severe forms. Among the severe forms, 40% (8/20) were due to mixed infections and 60% (12/20) to P. falciparum mono-infections. No mono-infections with other species were reported.

20- Besides it seems that mixed infections had less frequently respiratory distress. What were their severity criteria then?

**Response:** Other severity criteria were jaundice, severe anemia, impaired consciousness and hyperparasitemia.

21- L 366 13.18% should be written out in plain English, again in the next sentence

**Response:** These percentages have been written in plain letter, as well as in the following sentence.

22- L 367 change of which with of whom please. Do the authors refer 5.45% out of 13.18% or the whole population?

**Response:** The required correction has been made in the manuscript. The authors referred 5.45% out of the whole population (n=220).

23- Considering the small population one digit after coma would be enough please modify it throughout the manuscript

**Response:** We appreciate the reviewer's valuable feedback. Results have been presented in decimal as suggested by the reviewer.

24- L370-373 the authors could just send the reader to figure 6 without detailing all EBV serological results (conciseness)

**Response:** All details of EBV serological results have been removed and readers have been referred to figure 6.

25- L379 Please remove P>0.05. How did the authors attribute symptoms to EBV infection? do they refer instead to EBV serological profile?

**Response:** P>0.05 has been deleted in the manuscript as suggested by the reviewer. Symptoms of EBV have been attributed according to the symptoms listed in the literature review. After analyzing the symptoms, we tried to match them with EBV serological profile. Generally, EBV symptoms include fatigue, heart palpitations, cough, fever, sore throat, angina, inflamed throat, swollen lymph nodes in the neck, enlarged spleen, swollen liver, rash (<https://www.cdc.gov/epstein-barr/about-ebv.html>).

26- L384-387 should be in methods section. the authors refer to « high proportion and latter to « relatively high » L 420, please avoid it and just give raw and objective data in the results section

**Response:** L384-387 sentences have been deleted. “Relatively” has been deleted from the sentence.

27- L 389-390 please give number and frequencies to get clearly what do these percentages refer to. Please do it throughout the manuscript.

**Response:** We value the insightful comments provided by the reviewer. Numbers and frequencies have been added to the percentages described in the manuscript.

28- L 390-392 please reverse the sentence for clarity.

**Response:** The required correction has been made.

29- Fig 7 columns in grey levels in black and white are quite hard to distinguish here, please change for black or white columns. Moreover, according to me the histograms don’t represent the title of the figure. SM occur for 8/12 66% subjects and uncomplicated M in 4/12 33% for EBV reactivation group while SM occur for 12/38 32% subjects and uncomplicated M in 26/38 68% for EBV reactivation. The histograms should give these percentages instead of the current ones in my opinion.

**Response:** We extend our sincere appreciation to the reviewer for providing valuable insights into the methodology. Figure 7 has been redone. The columns in figure 7 were represented in black and white as suggested by the reviewer.

30- L398-400 should be in methods section

**Response:** The sentence has been deleted.

31- L 402 -403 What does malaria symptoms refer to here? please specify.

**Response:** They refer to the following symptoms: Fever, Fatigue, Sore muscle, Sweat and thrill, Anorexia, Pallor, Headaches, Vomiting, Nausea, Jaundice, Dizziness, Abdominal, Pain, Respiratory distress and anemia. These symptoms have been represented in figure S1 of the supporting information.

32- L558 560 it is not clear if the level of antibodies was assessed only in patients with positive results or if patients with negative results were also analysed here ? here it would to lead to compare both different levels of antibodies and positive versus negative results.
I think that this part of analysis on the levels of antibodies does not add to the study and the fundings of such « quantitative » analysis appear a little bit weak.

**Response:** We appreciate the constructive feedback from the reviewer regarding the EBV antibodies analysis. Antibody levels were assessed in patients with positive and negative results. We wanted to see if there was a correlation between antibody levels in positive and negative patients and malaria status (severe, uncomplicated or negative). This is the first time it has been shown that EBV antibody levels can be related to malaria severity. It also reflects the reactivation process of the virus during malaria. But more studies are needed.
