# Supplementary material for: Association between Epstein-Barr virus reactivation and severe malaria in pregnant women living in a malaria-endemic region of Cameroon
Source: PLOS Glob Public Health. 2024 Aug 12;4(8):e0003556. doi: 10.1371/journal.pgph.0003556 (PMC11318859; doi:10.1371/journal.pgph.0003556)
Supplement: Attachment — Submitted filename: Response to Reviewers.docx [file pgph.0003556.s005.docx]

To,

**Dr. Hugh Cowley**
Staff Editor
PLOS Global Public Health

Dear Dr. **Hugh Cowley**,

Thank you very much for considering our research article entitled **“****Association between Epstein-Barr virus reactivation and severe malaria in pregnant women living in a Malaria-endemic region of Cameroon*”*** by Djuidje Chatue et al., for revision. The authors appreciate and extend thanks for the kind consideration and thorough review of this manuscript that allowed us to revise and improve the manuscript. Keeping the reviewers helpful and validly raised concerns and questions in mind we modified and improved the manuscript.

We have addressed the questions raised or corrections suggested by the reviewer point by point. Please find the details below.

Best wishes and regards

DJUIDJE CHATUE Ide Armelle

Ph.D. Student in Medical Microbiology, University of Yaounde I

**Journal Requirements:**

1. Please review your reference list to ensure that it is complete and correct. If you have cited papers that have been retracted, please include the rationale for doing so in the manuscript text, or remove these references and replace them with relevant current references. Any changes to the reference list should be mentioned in the rebuttal letter that accompanies your revised manuscript. If you need to cite a retracted article, indicate the article’s retracted status in the References list and also include a citation and full reference for the retraction notice.

**Response:** The list of references has been updated. We have carefully checked that they are complete and correct. No reproduced articles have been cited in the manuscript.

2. We ask that a manuscript source file is provided at Revision. Please upload your manuscript file as a .doc, .docx, .rtf or .tex.

**Response:** The manuscript source file will be provided in .doc format at the submission.

**Reviewer Comments to Author:**

**Reviewer #3:**

**Comments:** The authors set out to determine whether there is an association between the reactivation of EBV and the occurrence of severe malaria in pregnant women residing in a meso-endemic malaria zone of Cameroon. To answer this research question they conducted a cross-sectional hospital-based study in three locations in the west region of Cameroon. Using a structured questionnaire they recruited 220 consenting pregnant women, from whom they collected blood for diagnosis of malaria by microscopy and PCR and for preparation of plasma for serology of EBV. Reactivation of EBV was done by measuring IgM specific antibodies whereas IgG antibodies detection signified exposure. As expected more than 95 % of the participants were exposed to EBV. Severe malaria was significantly more prevalent in the subgroup of participants with reactivated EBV as compared to those exposed to latent EBV infection.

1- Severe malaria is usually more prevalent in patients with mega-parasitaemia in a hyper-endemic area. The authors should explain how severe malaria was diagnosed and discuss the relatively low incidence of severe malaria in their study.

**Response:** We extend our sincere appreciation to the reviewer for acknowledging our effort in this work and for raising this relevant concern. As you mentioned, severe malaria is more common in patients with hyperparasitemia in a hyperendemic area. It’s well known that hyperparasitemia is a criteria for severe *Plasmodium falciparum* malaria, according to the World Health Organization (WHO). However, the definition of hyperparasitemia may not be similar in different regions. In areas of high stable malaria transmission, the WHO has defined hyperparasitemia as >5% or 250,000/μL, and in areas of low transmission as >2%/100,000/μL. In the present study, severe malaria was diagnosed by the presence of asexual forms of the *Plasmodium* parasite in thick blood smear, determination of parasite density, and the presence of at least one biological or clinical feature described by the WHO, including prostration, Altered consciousness: modified Glasgow Coma Scale <10; Repeated generalized seizures (more than 2 in 24 hours); Respiratory distress; Pulmonary edema: radiological definition; Shock state (systolic blood pressure < 80 mmHg and peripheral signs of circulatory failure); Abnormal bleeding; Hemoglobinuria (dark red urine, hemoglobinuria on dipstick); Renal failure (creatinine > 265 µmol/l and/or oliguria <400 ml/day); Hypoglycemia (blood glucose < 2.2 mmol/l); Severe anemia (Hb < 5g/dl or Ht <15%); Acidosis: bicarbonate < 15 mmol/l ± pH < 7.35; Hyperlactatemia: plasma lactate > 5 mmol/l; Hyperparasitemia = 4% in non-immune; Jaundice (clinical or total bilirubin > 50 µmol/l). Therefore, pregnant women with hyperparasitemia > 100,000/μL associated with either jaundice, respiratory distress, profound anemia < 5g/dl, and/or altered consciousness were classified as having severe malaria.

**Reference:** (1) World Health Organization. Guidelines for the treatment of malaria. 2nd ed. Geneva: World Health Organization; 2010, p. 35; (2) Wilairatana, Polrat & Tangpukdee, Noppadon & Krudsood, Srivicha. (2013). Definition of hyperparasitemia in severe *falciparum malaria* should be updated. Asian Pacific Journal of Tropical Biomedicine. 3. 586. 10.1016/S2221-1691(13)60119-7.).

Regarding the relatively low incidence of severe malaria in this study, it may be due to the moderate transmission of malaria in the region. Previous studies conducted in several cities in the West region of Cameroon have reported malaria prevalence among pregnant women of 53.4%, 25%, and 25.3%, respectively, in the towns of Foumbot (Ngouyamsa N. A. Sidiki et al), Bafang (Christian Taheu N. et al 2022), and Dschang (Kamga et al 2023). In this study, we found a prevalence of 23.2% in the Bangangte and Bafoussam. Furthermore, 64.1% of pregnant women had taken at least one dose of IPT, and 60.5% were using LLINs at the time of the study, which may have also contributed to the decrease in the burden of severe malaria.

2- There were a few grammatical slips which could be corrected by careful proof reading eg: line 145 : Write 'study sites.' instead of 'study site'.

p228: Give the key of (+), (-), (+/-) in a legend under Table 1.

line 269: There is no ethnic group in Cameroon called Bamenda. Do you mean people from the North West region?

line 365: Write "We assessed.." instead of "we assess..."

line 388: Write, "First of its kind.." instead of "first of its.."

line 390: Do not capitalize 'dendritic'

line 423: Write "However..."instead of "On the other hand..."

**Response:** We thank the reviewer for providing valuable insights. The required corrections have been made in the manuscript.
